# Supplementary material for: Single-Cell Epigenomics Uncovers Heterochromatin Instability and Transcription Factor Dysfunction during Mouse Brain Aging
Source: bioRxiv. 2025 May 20:2025.04.21.649585. Preprint. [Version 2] doi: 10.1101/2025.04.21.649585 (PMC12139859; doi:10.1101/2025.04.21.649585)
Supplement: Supplement 7 [file NIHPP2025.04.21.649585v2-supplement-7.pdf]

# Supplementary Figures

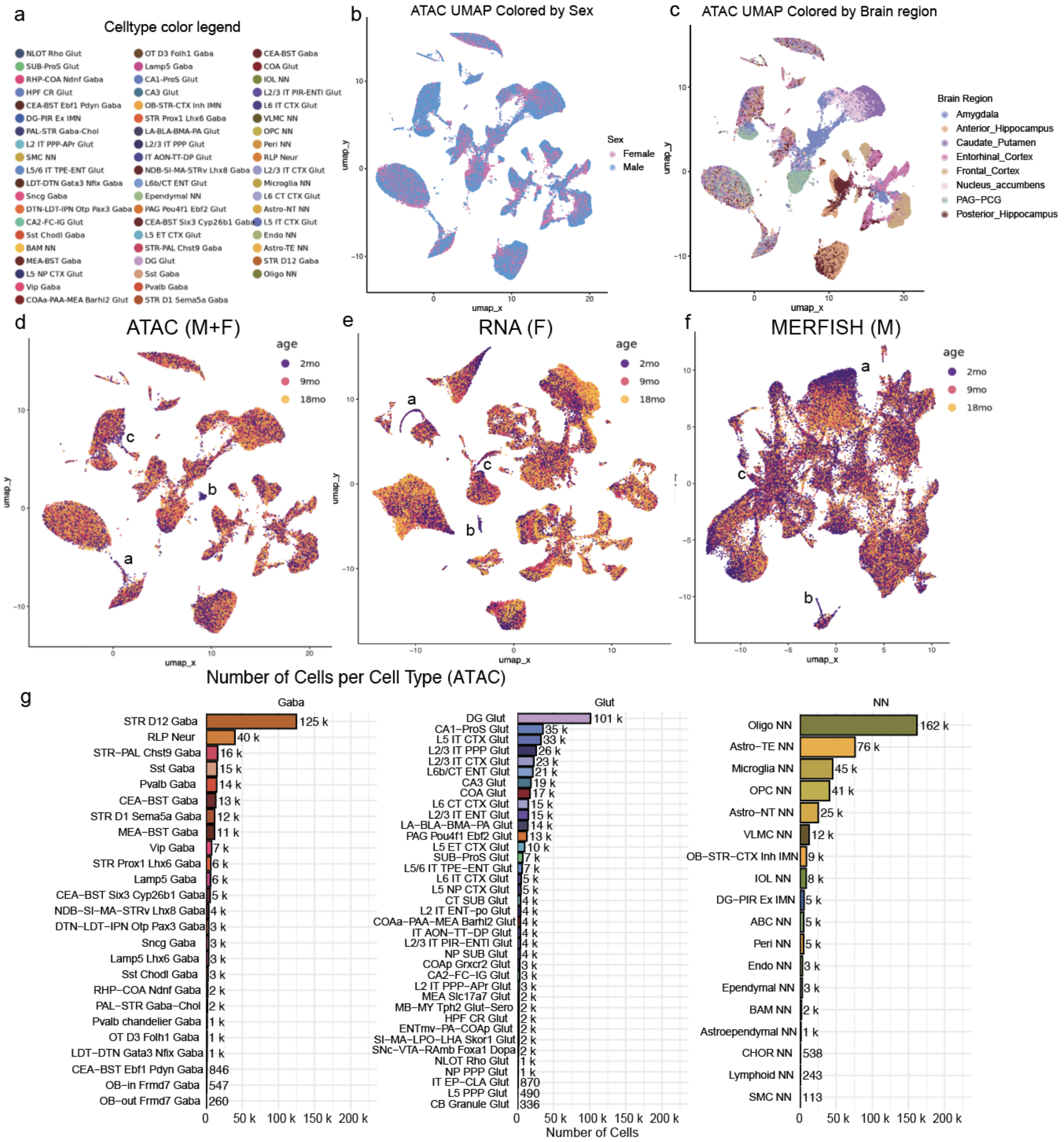


**Figure S1. Characterization of multi-omic datasets and age-related gene regulation.**
(A) Cell type color legend corresponding to Figures 1B and 2A.

bioRxiv preprint doi: https://doi.org/10.1101/2025.04.21.649585; this version posted May 20, 2025. The copyright holder for this preprint (which was not certified by peer review) is the author/funder, who has granted bioRxiv a license to display the preprint in perpetuity. It is made available under aCC-BY 4.0 International license.

(B) UMAP of snATAC-seq data annotated by sex.
(C) UMAP of snATAC-seq data annotated by brain region.
(D-F) UMAP projections of scATAC-seq, scRNA-seq, and MERFISH data colored by age group (2mo, 9mo, 18mo).
(G) Number of cells per celltype for ATAC data (male and female)

bioRxiv preprint doi: https://doi.org/10.1101/2025.04.21.649585; this version posted May 20, 2025. The copyright holder for this preprint (which was not certified by peer review) is the author/funder, who has granted bioRxiv a license to display the preprint in perpetuity. It is made available under aCC-BY 4.0 International license.

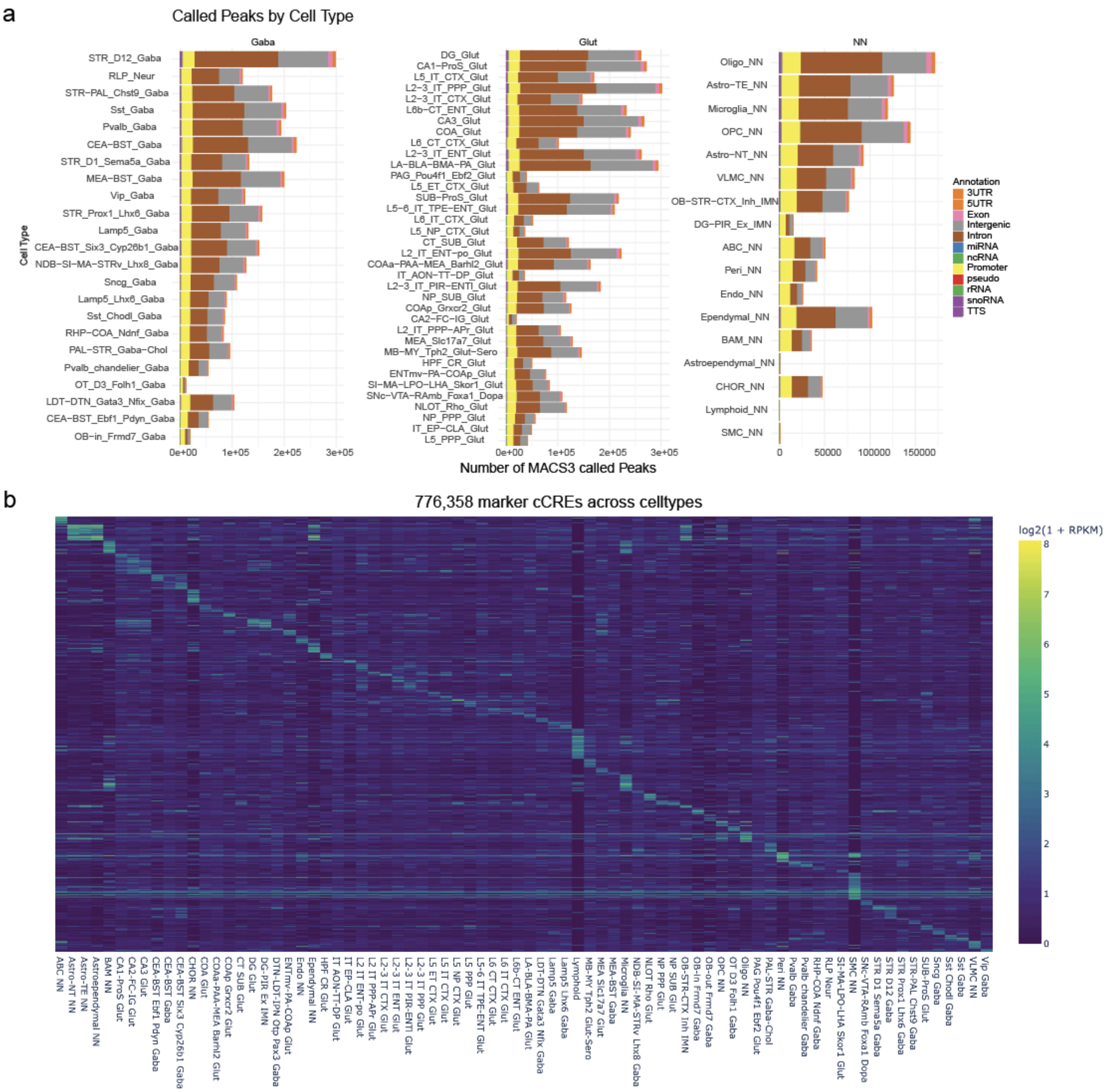


**Figure S2. Cell type-level peak calling identifies cell-type-specific regulatory signatures.**
(A) Number of peaks called by MACS3 per cell type, colored by genomic annotation.
(B) Heatmap showing accessibility ($log_2$[1 + RPKM]) at marker cis-regulatory elements (cCREs) across cell types.

bioRxiv preprint doi: https://doi.org/10.1101/2025.04.21.649585; this version posted May 20, 2025. The copyright holder for this preprint (which was not certified by peer review) is the author/funder, who has granted bioRxiv a license to display the preprint in perpetuity. It is made available under aCC-BY 4.0 International license.

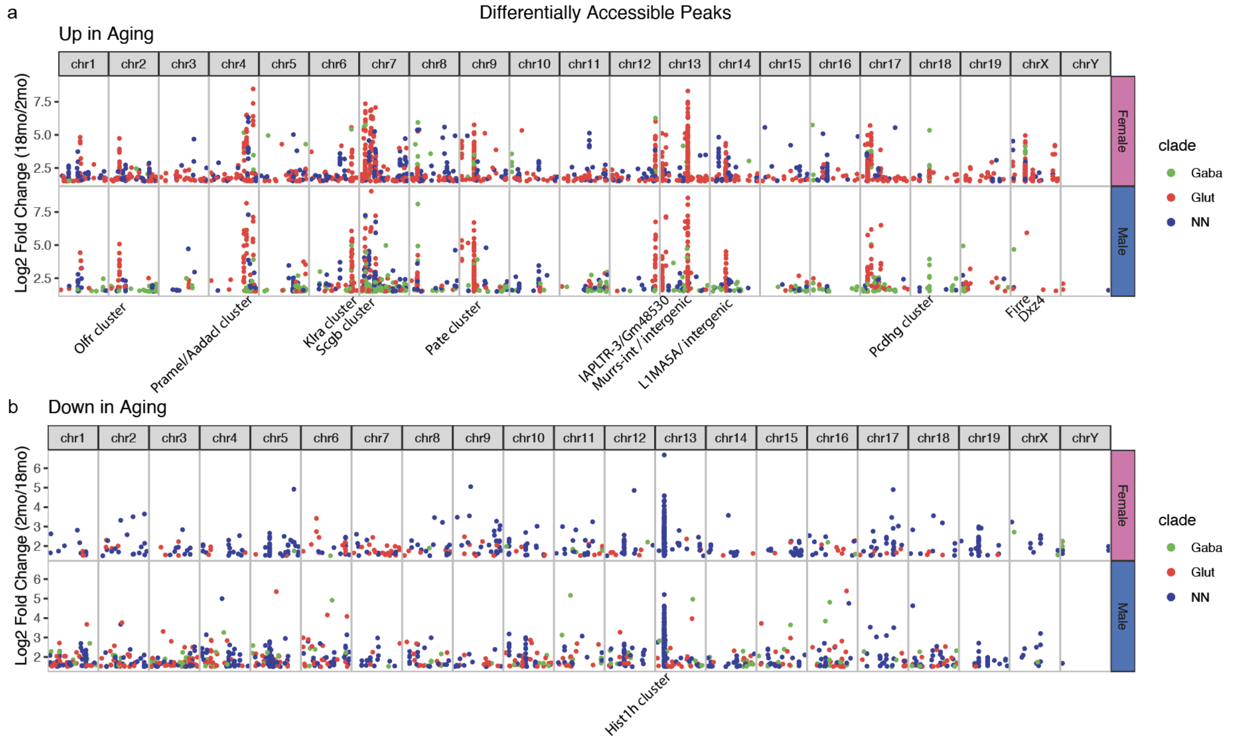


**Figure S3. Genome-wide locations of age-associated chromatin accessibility changes in male and female brains.**
(A) Genomic locations of age-upregulated differentially accessible regions (DARs), plotted as $\log_2$ fold change (18mo/2mo) across chromosomes.
(B) Genomic locations of age-downregulated DARs, plotted as $\log_2$ fold change (2mo/18mo) across chromosomes.

bioRxiv preprint doi: https://doi.org/10.1101/2025.04.21.649585; this version posted May 20, 2025. The copyright holder for this preprint (which was not certified by peer review) is the author/funder, who has granted bioRxiv a license to display the preprint in perpetuity. It is made available under aCC-BY 4.0 International license.

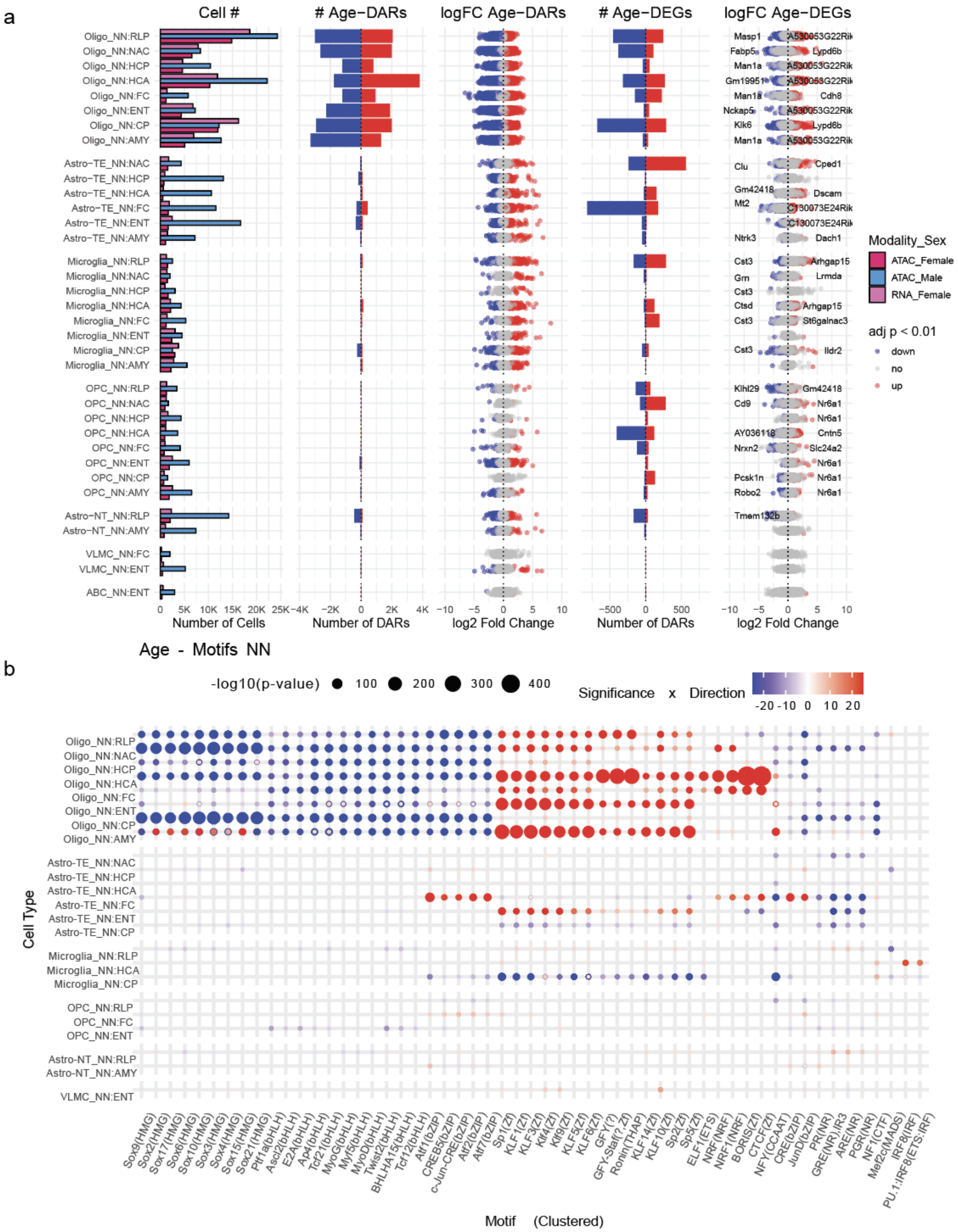


**Figure S4. Aging-associated chromatin and transcriptional changes in non-neuronal cell types.**

(A) Left to right: Cell counts per non-neuronal cell type, brain region, and modality. Bar plot showing the number of age-associated DARs (adj. p < 0.01, $|log_2FC| > 0.25$). Scatter plots of

bioRxiv preprint doi: https://doi.org/10.1101/2025.04.21.649585; this version posted May 20, 2025. The copyright holder for this preprint (which was not certified by peer review) is the author/funder, who has granted bioRxiv a license to display the preprint in perpetuity. It is made available under aCC-BY 4.0 International license.

$\log_2$ fold change ($\log_2$FC) for DARs, colored by direction (up- or downregulated with age). Bar plot showing the number of age-associated DEGs (adj. $p < 0.01$, $|\log_2\text{FC}| > 0.25$).
(B) Motif enrichment in age-DARs across non-neuronal cell types. Dot size indicates $-\log_{10}$(p-value), and color denotes direction of change with aging.

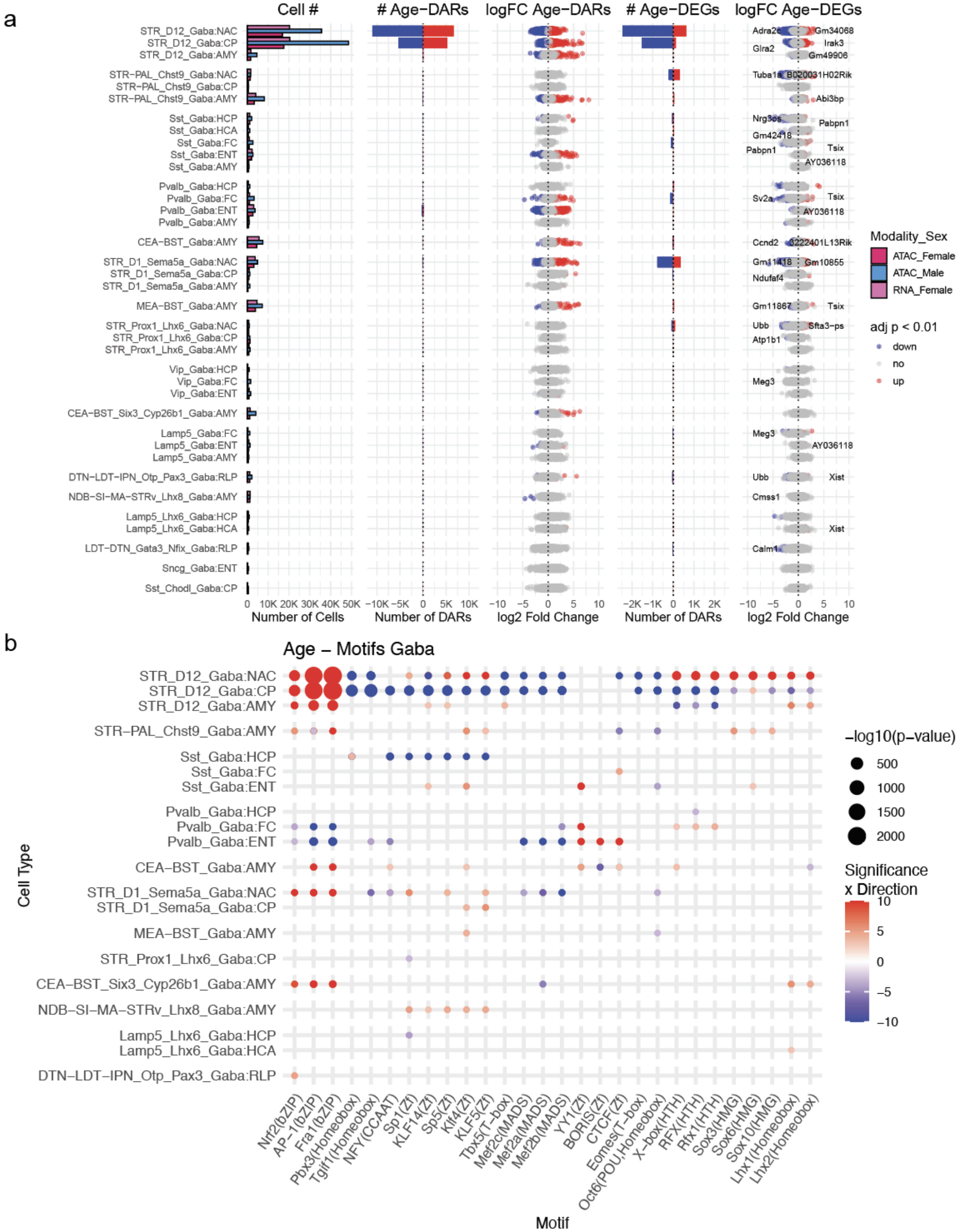

bioRxiv preprint doi: https://doi.org/10.1101/2025.04.21.649585; this version posted May 20, 2025. The copyright holder for this preprint (which was not certified by peer review) is the author/funder, who has granted bioRxiv a license to display the preprint in perpetuity. It is made available under aCC-BY 4.0 International license.

**Figure S5. Aging-associated chromatin and transcriptional changes in GABAergic cell types.**
(A) Left to right: Cell counts per GABAergic cell type, brain region, and modality. Bar plot showing the number of age-associated DARs (adj. $p < 0.01$, $|\log_2 FC| > 0.25$). Scatter plots of $\log_2$ fold change ($\log_2 FC$) for DARs, colored by direction (up- or downregulated with age). Bar plot showing the number of age-associated DEGs (adj. $p < 0.01$, $|\log_2 FC| > 0.25$). Scatter plots of $\log_2 FC$ for DEGs, colored by direction of change.
(B) Motif enrichment in age-DARs across GABAergic cell types. Dot size indicates $-\log_{10}$(p-value), and color denotes direction of change with aging.

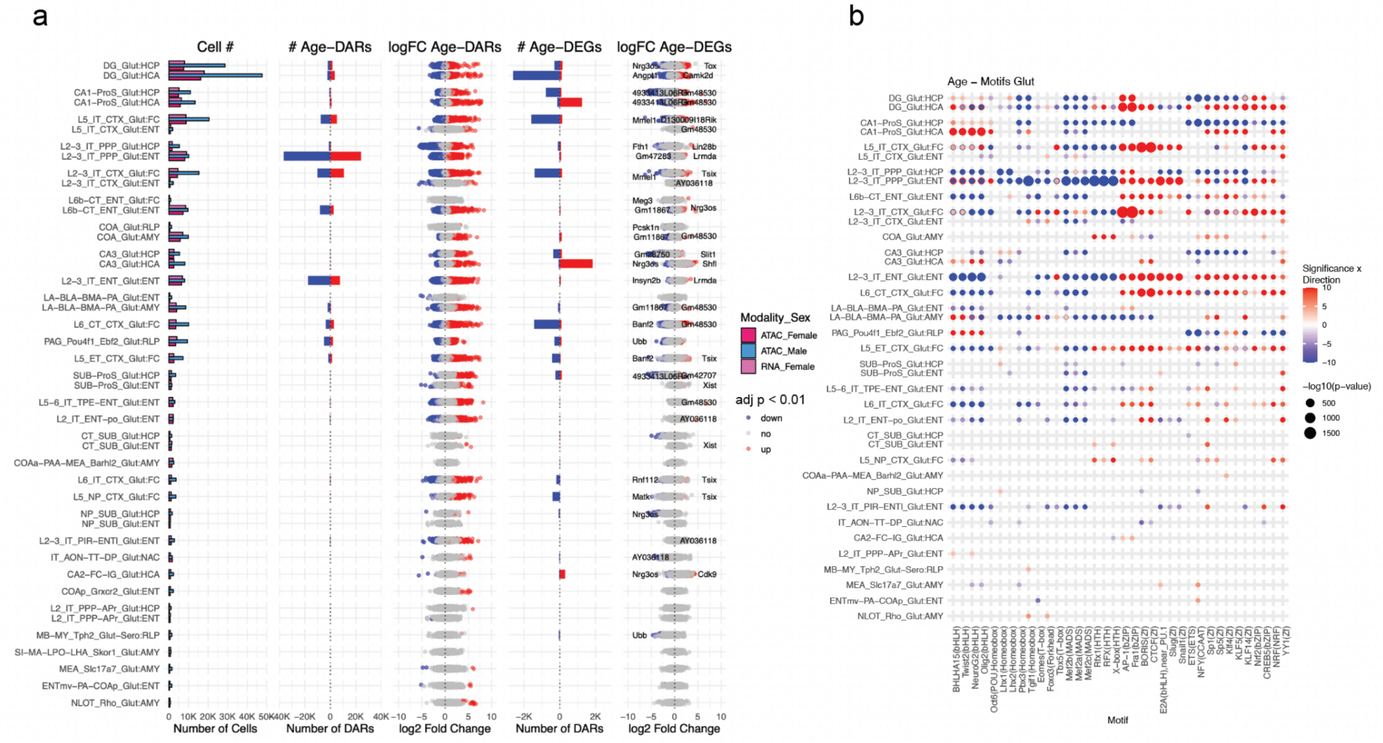


**Figure S6. Aging-associated chromatin and transcriptional changes in glutamatergic cell types.**
(A) Left to right: Cell counts per glutamatergic cell type, brain region, and modality. Bar plot showing the number of age-associated DARs (adj. $p < 0.01$, $|\log_2 FC| > 0.25$). Scatter plots of $\log_2$ fold change ($\log_2 FC$) for DARs, colored by direction (up- or downregulated with age). Bar plot showing the number of age-associated DEGs (adj. $p < 0.01$, $|\log_2 FC| > 0.25$). Scatter plots of $\log_2 FC$ for DEGs, colored by direction of change.
(B) Motif enrichment in age-DARs across glutamatergic cell types. Dot size indicates $-\log_{10}$(p-value), and color denotes direction of change with aging.

bioRxiv preprint doi: https://doi.org/10.1101/2025.04.21.649585; this version posted May 20, 2025. The copyright holder for this preprint (which was not certified by peer review) is the author/funder, who has granted bioRxiv a license to display the preprint in perpetuity. It is made available under aCC-BY 4.0 International license.

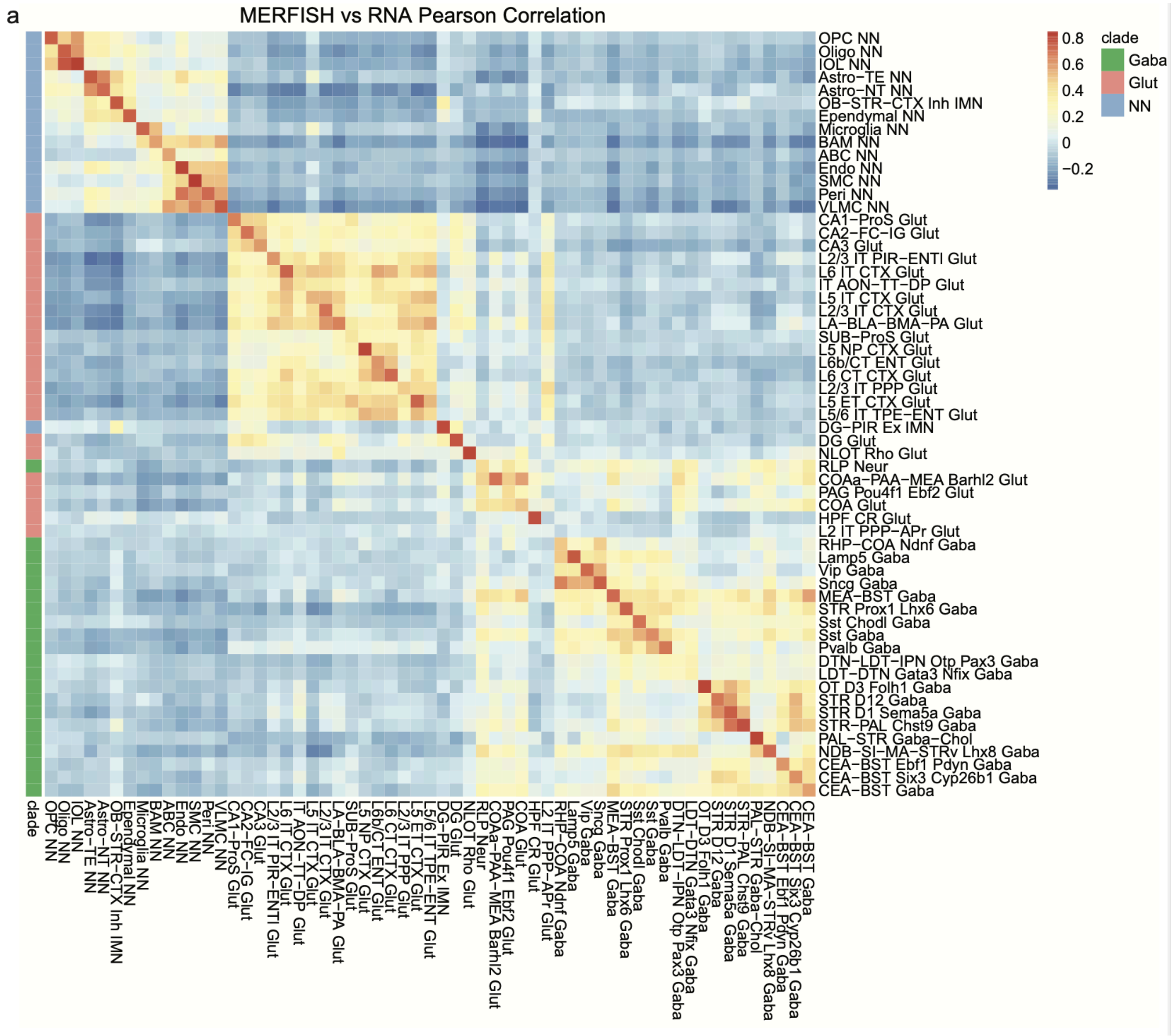


**Figure S7. Cross-modal correlation of gene expression across cell types.**
(A) Heatmap showing Pearson correlation coefficients between RNA-seq and MERFISH gene expression profiles across shared cell types. Expression values were CLR-normalized per cell, aggregated by cell type, and RNA-seq values were additionally normalized by gene length. Correlations were computed across shared genes to assess concordance between modalities.

bioRxiv preprint doi: https://doi.org/10.1101/2025.04.21.649585; this version posted May 20, 2025. The copyright holder for this preprint (which was not certified by peer review) is the author/funder, who has granted bioRxiv a license to display the preprint in perpetuity. It is made available under aCC-BY 4.0 International license.

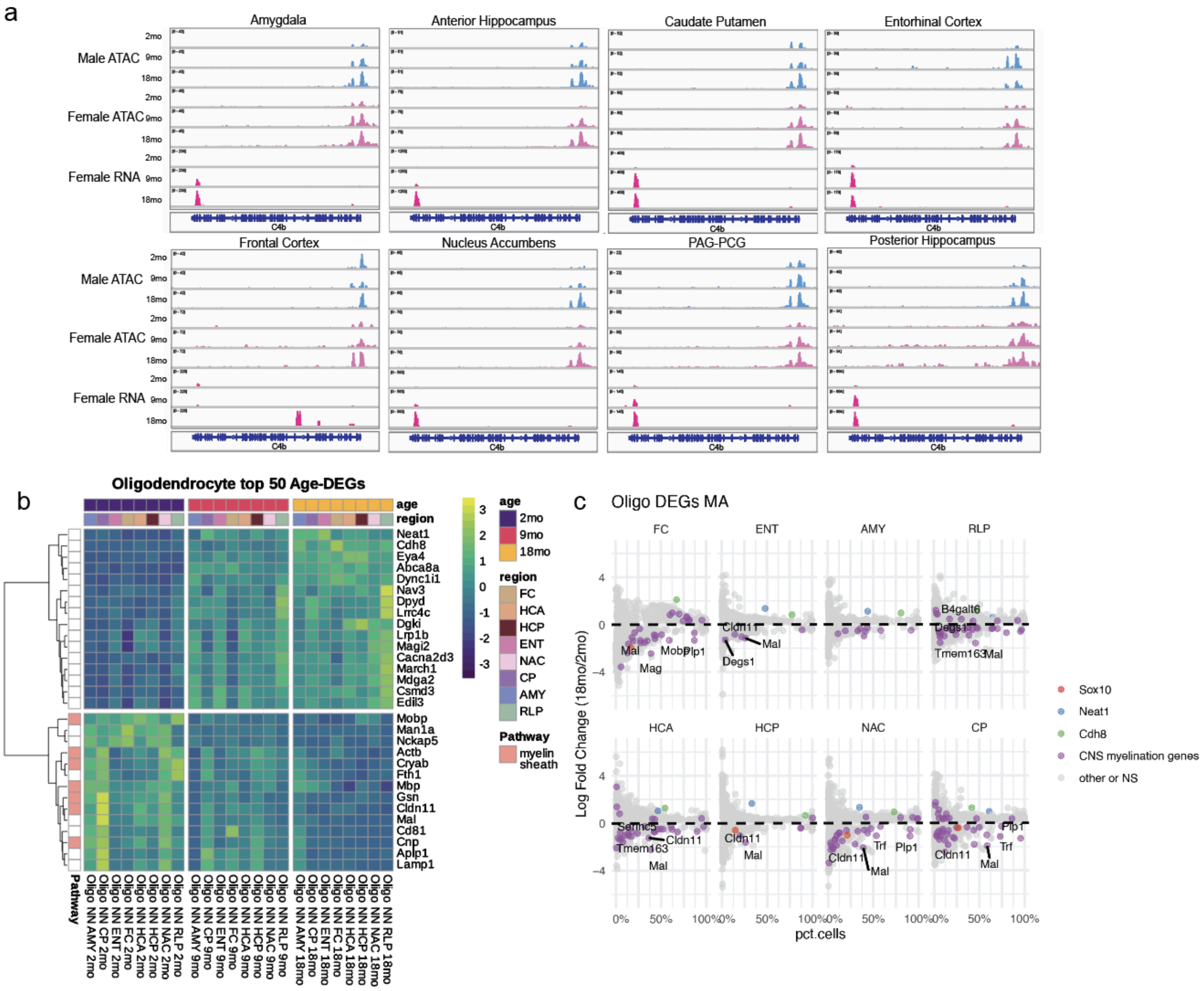


# Figure S8. Downregulation of myelination-associated genes in aging oligodendrocytes.

(A) Genome browser tracks showing chromatin accessibility (male ATAC, female ATAC) and RNA expression (female RNA) across age in eight brain regions in oligodendrocytes at the age-associated gene C4b.
(B) Heatmap of the top 30 age-associated DEGs in oligodendrocytes, showing consistent downregulation of genes enriched for the “myelin sheath” GO term across brain regions.
(C) MA plots of DEGs in oligodendrocytes, highlighting myelination-related genes (*Mal*, *Mog*, *Olig1*). X-axis denotes the percentage of cells expressing each gene; Y-axis shows $\log_2$ fold change (18mo vs. 2mo).

bioRxiv preprint doi: https://doi.org/10.1101/2025.04.21.649585; this version posted May 20, 2025. The copyright holder for this preprint (which was not certified by peer review) is the author/funder, who has granted bioRxiv a license to display the preprint in perpetuity. It is made available under aCC-BY 4.0 International license.

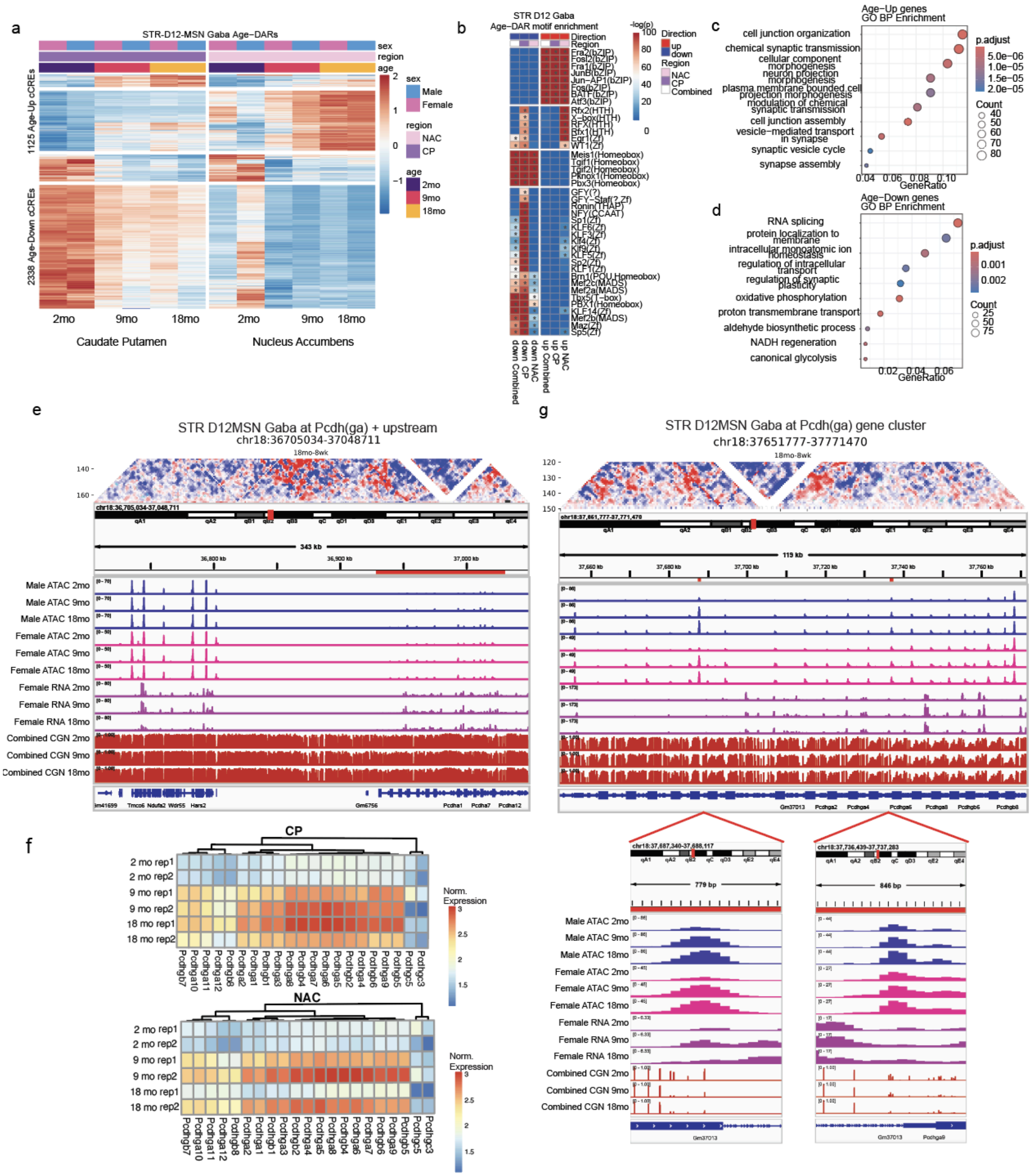


**Figure S9. Aging-associated chromatin remodeling and protocadherin locus reorganization in D12 MSN neurons.**
(A) Heatmap showing scaled, normalized chromatin accessibility at age-differential cCREs in D12 medium spiny neurons (MSNs) from the striatum.
(B) Motif enrichment analysis for age-upregulated and age-downregulated cCREs in D12 MSNs.
(C) MA plot of age-differentially expressed genes (DEGs) in D12 MSNs (18mo vs. 2mo).
(D) GO Biological Process enrichment for age-upregulated (left) and age-downregulated (right) DEGs.

bioRxiv preprint doi: https://doi.org/10.1101/2025.04.21.649585; this version posted May 20, 2025. The copyright holder for this preprint (which was not certified by peer review) is the author/funder, who has granted bioRxiv a license to display the preprint in perpetuity. It is made available under aCC-BY 4.0 International license.

(E) Genome browser view of the *Protocadherin gamma* (Pcdhg) gene cluster in D12 MSNs showing increased chromatin accessibility, gene expression, hypomethylation, and altered Hi-C contact frequency with aging.
(F) Heatmap of normalized expression levels for *Pcdhg* genes across age groups and brain regions in D12 MSN GABAergic neurons.
(G) Zoomed-in IGV view of the *Pcdhg* locus highlighting specific aging-associated regulatory changes.

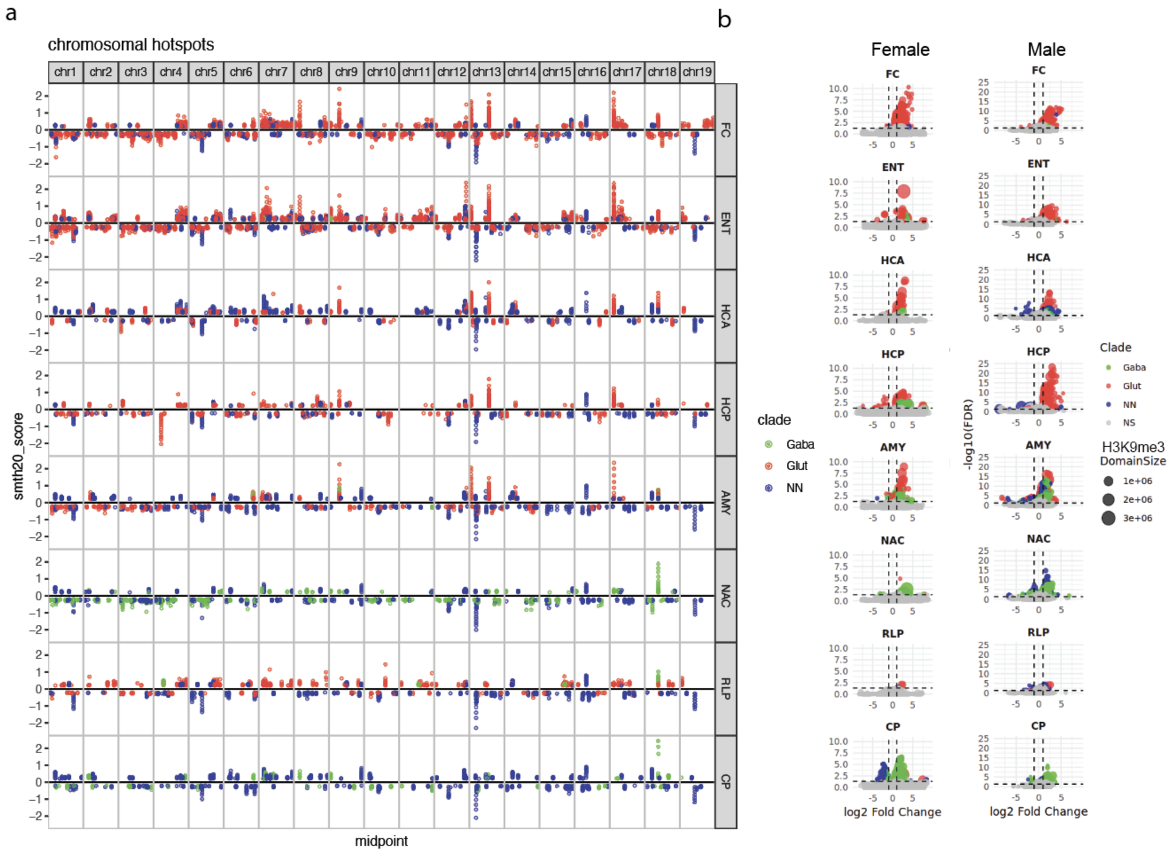


**Figure S10. Chromosomal aging hotspots and heterochromatin loss across brain regions.**
(A) Chromosomal aging hotspots with significantly more DARs than expected by chance across cell types. Points are colored by cell type clade (glutamatergic, GABAergic, non-neuronal) and faceted by brain region, highlighting regions of age-associated chromatin accessibility gain or loss.
(B) Volcano plots of differential chromatin accessibility within H3K9me3-marked domains across brain regions for glutamatergic, GABAergic, and non-neuronal cells. X-axis: $\log_2$ fold change (18mo/2mo); Y-axis: $-\log_{10}$(adjusted p-value). Point size represents the length of the H3K9me3 domain.

bioRxiv preprint doi: https://doi.org/10.1101/2025.04.21.649585; this version posted May 20, 2025. The copyright holder for this preprint (which was not certified by peer review) is the author/funder, who has granted bioRxiv a license to display the preprint in perpetuity. It is made available under aCC-BY 4.0 International license.

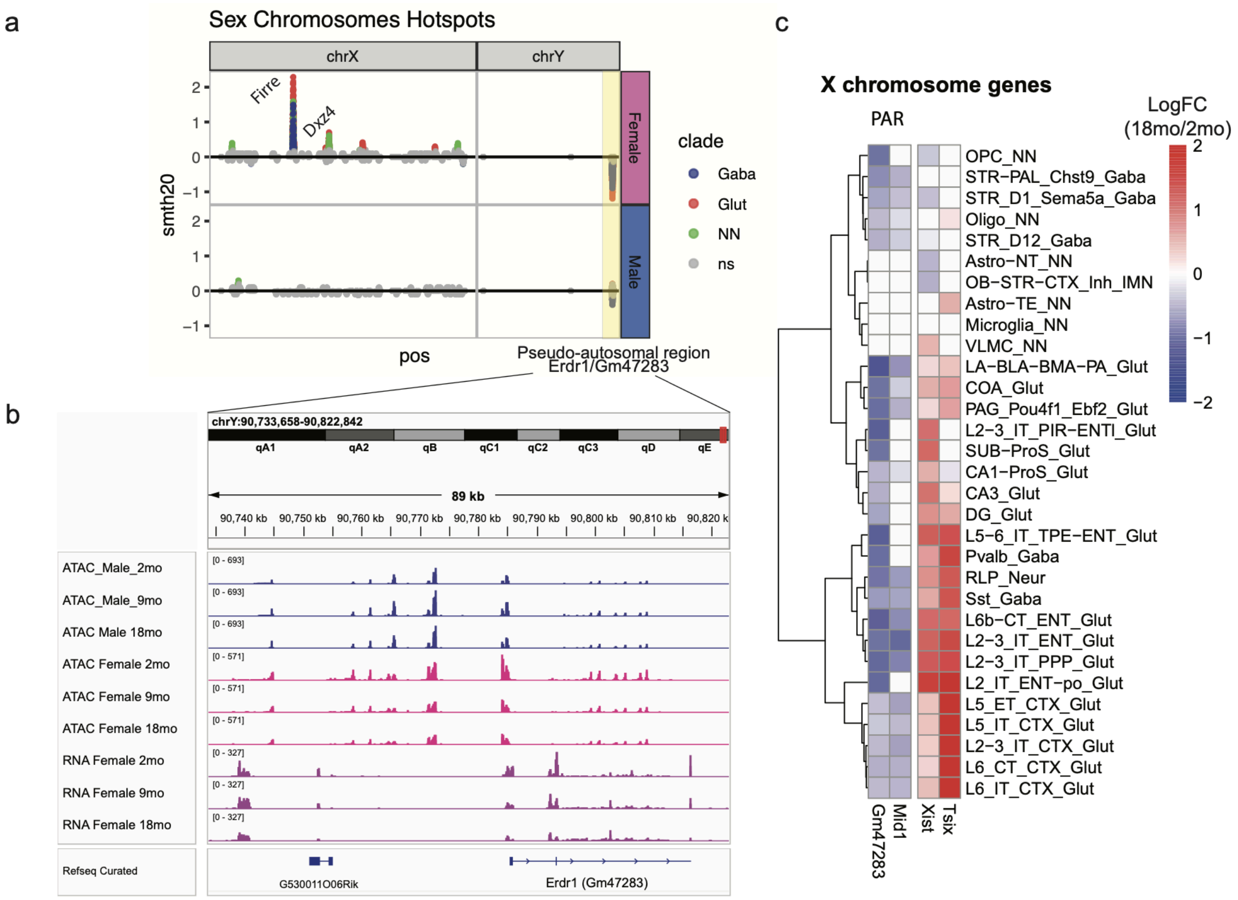


**Figure S11. Sex chromosome aging hotspots and pseudoautosomal region remodeling.**
(A) Aging-associated chromatin accessibility hotspots on the sex chromosomes. Highlighted region at the distal end of chromosome Y corresponds to the pseudoautosomal region.
(B) Genome browser view (IGV) of the pseudoautosomal region containing *Erdr1* (also known as *Gm47283*) across representative cell type STR_D12_GABA, showing chromatin accessibility (male ATAC, female ATAC) and RNA expression (female RNA) across age groups.
(C) Heatmap of age-related $\log_2$ fold change (18mo/2mo) in expression for pseudoautosomal region genes (*Gm47283*, *Mid1*) and X-inactivation regulators (*Xist*, *Tsix*) across major cell types.

bioRxiv preprint doi: https://doi.org/10.1101/2025.04.21.649585; this version posted May 20, 2025. The copyright holder for this preprint (which was not certified by peer review) is the author/funder, who has granted bioRxiv a license to display the preprint in perpetuity. It is made available under aCC-BY 4.0 International license.

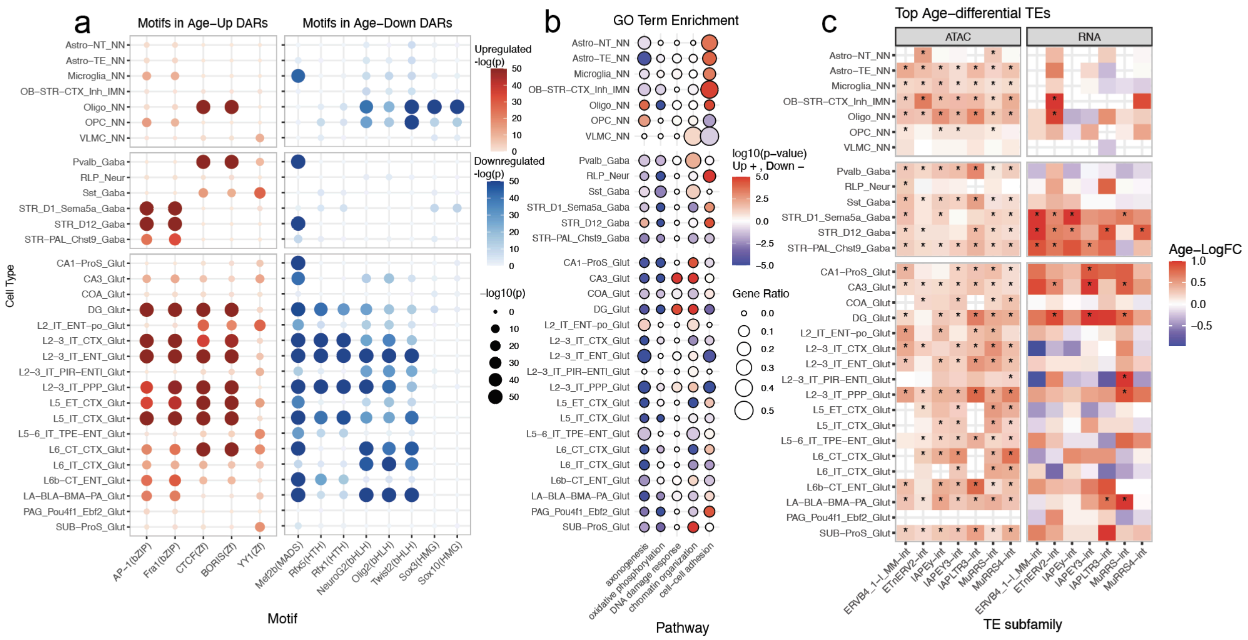


**Figure S12. Top aging-associated regulatory and transcriptional changes across cell types.**
(A) Motif enrichment analysis of age-associated differentially accessible regions (age-DARs), showing top enriched transcription factor motifs across cell types.
(B) Gene Ontology (GO) pathway enrichment analysis of age-associated differentially expressed genes (age-DEGs), highlighting recurrent biological processes altered with aging.
(C) Top differentially accessible transposable element (TE) subfamilies across aging, ranked by frequency and magnitude of accessibility changes.

bioRxiv preprint doi: https://doi.org/10.1101/2025.04.21.649585; this version posted May 20, 2025. The copyright holder for this preprint (which was not certified by peer review) is the author/funder, who has granted bioRxiv a license to display the preprint in perpetuity. It is made available under aCC-BY 4.0 International license.

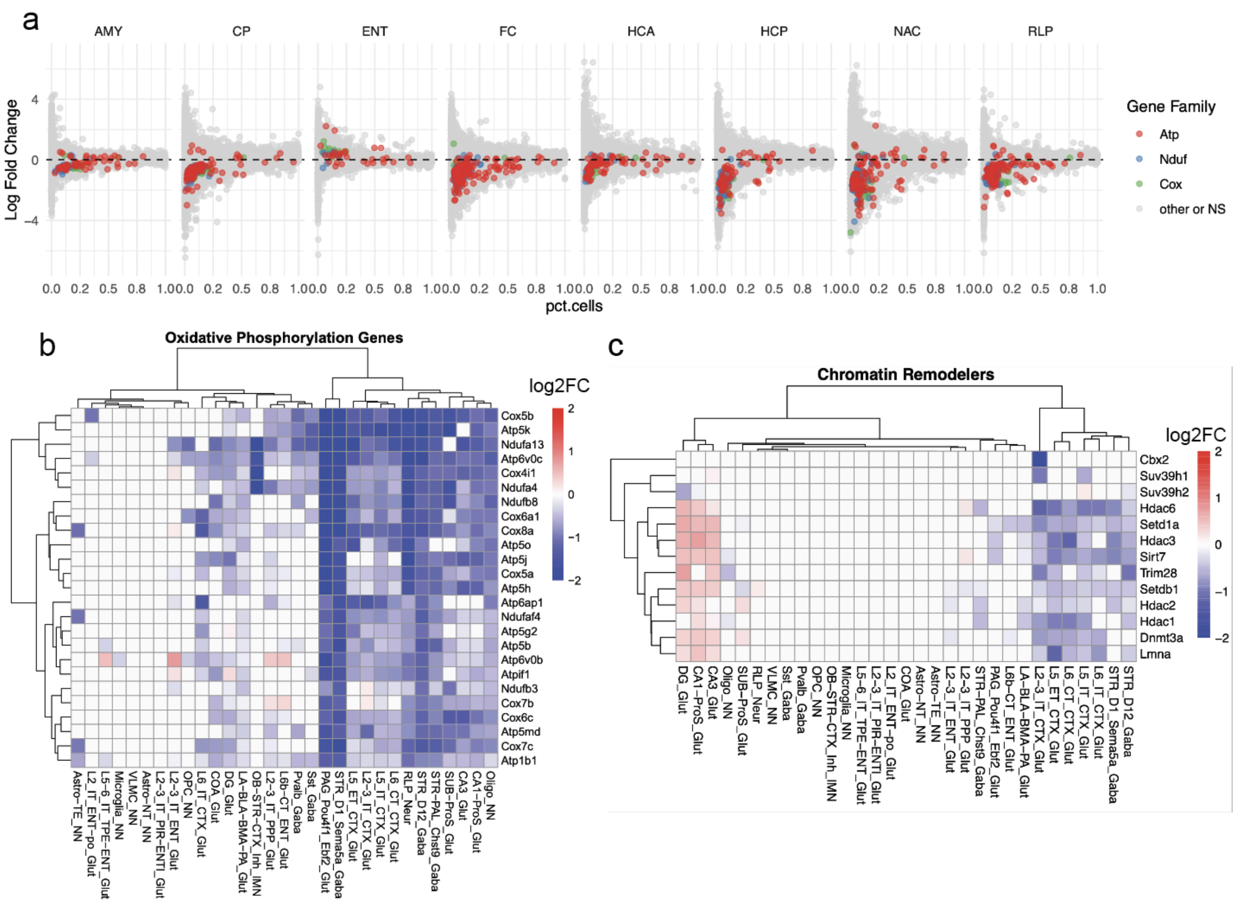


**Figure S13. Aging-associated downregulation of oxidative phosphorylation and heterochromatin maintenance genes.**
(A) MA plots highlighting age-associated downregulated genes (18mo vs. 2mo) related to oxidative phosphorylation across brain regions.
(B) Heatmap showing $\log_2$ fold change ($\log_2$FC) in expression of top oxidative phosphorylation-related genes across major cell types.
(C) Heatmap showing $\log_2$FC in expression of genes involved in heterochromatin maintenance, indicating widespread transcriptional decline of chromatin regulators with aging.

**Supplementary tables**

Table S1. Brain Dissection Region Annotation
Table S2. MERFISH gene panel
Table S3. ATAC Metadata
Table S4. RNA Metadata
Table S5. MERFISH Metadata
Table S6. cCREs called by celltype table
